# Therapies Targeted to Androgen Receptor Signaling Axis in Prostate Cancer: Progress, Challenges, and Hope

**DOI:** 10.3390/cancers12010051

**Published:** 2019-12-23

**Authors:** Sirin Saranyutanon, Sanjeev Kumar Srivastava, Sachin Pai, Seema Singh, Ajay Pratap Singh

**Affiliations:** 1 Department of Pathology, College of Medicine, University of South Alabama, Mobile, AL 36617, USA; ss1830@jagmail.southalabama.edu (S.S.);; 2 Department of Oncologic Sciences, Mitchell Cancer Institute, University of South Alabama, Mobile, AL 36604, USA; 3 Department of Medical Oncology, Mitchell Cancer Institute, University of South Alabama, Mobile, AL 36604, USA; spai@health.southalabama.edu; 4 Department of Biochemistry and Molecular Biology, College of Medicine, University of South Alabama, Mobile, AL 36688, USA

**Keywords:** prostate cancer, androgens, androgen receptor signaling

## Abstract

Prostate cancer is the mostly commonly diagnosed non-cutaneous malignancy and the second leading cause of cancer-related death affecting men in the United States. Moreover, it disproportionately affects the men of African origin, who exhibit significantly greater incidence and mortality as compared to the men of European origin. Since androgens play an important role in the growth of normal prostate and prostate tumors, targeting of androgen signaling has remained a mainstay for the treatment of aggressive prostate cancer. Over the years, multiple approaches have been evaluated to effectively target the androgen signaling pathway that include direct targeting of the androgens, androgen receptor (AR), AR co-regulators or other alternate mechanisms that impact the outcome of androgen signaling. Several of these approaches are currently in clinical practice, while some are still pending further development and clinical evaluation. This remarkable progress has resulted from extensive laboratory, pre-clinical and clinical efforts, and mechanistic learnings from the therapeutic success and failures. In this review, we describe the importance of androgen signaling in prostate cancer biology and advances made over the years to effectively target this signaling pathway. We also discuss emerging data on the resistance pathways associated with the failure of various androgen signaling- targeted therapies and potential of this knowledge for translation into future therapies for prostate cancer.

## 1. Introduction

Prostate cancer (PCa) is a deadly malignancy that currently stands as the second leading cause of cancer-related death in American men. American Cancer Society estimates that about 174,650 men will be diagnosed with prostate cancer in 2019 and about 31,620 will die because of it [1]. In addition, significant race-related prostate cancer health disparities are also reported. Prostate cancer hits African Americans most disproportionately with nearly 60% greater incidence and more than two times greater mortality as compared to their European American counterparts [2,3]. Clinical management of prostate cancer includes surgery, radiation, and chemo- and targeted-therapies. In most patients, prostate cancer is diagnosed early at a localized stage and they undergo either surgery or radiation treatment or both. However, a significant proportion of cancer patients receives diagnosis at a stage when the tumor has already metastasized to the nearby or distant organs. In several others, prostate cancer recurs following surgery or radiotherapy and these metastatic and/or recurrent cancers require additional treatment options.

Androgens play an important role in the growth of prostate and prostate tumors [4]. Therefore, targeting of androgen signaling has remained a mainstay for the treatment of metastatic prostate cancer for past several decades [5]. Over the years, multiple approaches have been evaluated to effectively target the androgen signaling pathway that include direct targeting of the ligand (androgens), the receptor (androgen receptor; AR), AR co-regulators or other alternate mechanisms that impact the outcome of androgen signaling [6,7,8]. In the subsequent sections, we discuss the importance of androgen signaling in prostate cancer biology and advances made over the years to effectively target this signaling pathway of which several have already been brought into clinical practice. We also provide a brief discussion of the emerging data on the resistance pathways associated with the failure of androgen signaling-targeted therapies and potential of this knowledge for translation into future therapies for prostate cancer.

## 2. The Cellular and Molecular Progression of Prostate Cancer

The prostate is a walnut-sized gland in men that produces seminal fluid to nurture and transport the sperms. It is comprised of four zones; anterior fibromuscular stroma, a central zone, a peripheral zone, and the periurethral transition zone [9,10]. Most prostate carcinomas (~60–75%) arise from the bulkiest peripheral zone [9,10,11], whereas the transition zone has been suggested to be involved in the development of benign prostatic hyperplasia (BPH) [9]. Histologically, human prostate is composed of a pseudostratified epithelium and a stromal compartment that are separated by a basement membrane. The pseudostratified epithelium is characterized with the presence of three terminally differentiated epithelial cells viz. luminal, basal, and neuroendocrine cells [12]. Emerging data suggest that most PCa originate from either the luminal or basal cell types [13,14,15]. It is; however, a subject of debate whether prostate tumors originating from one cell type are more aggressive than those originating from others [16,17].

**Figure 1:**
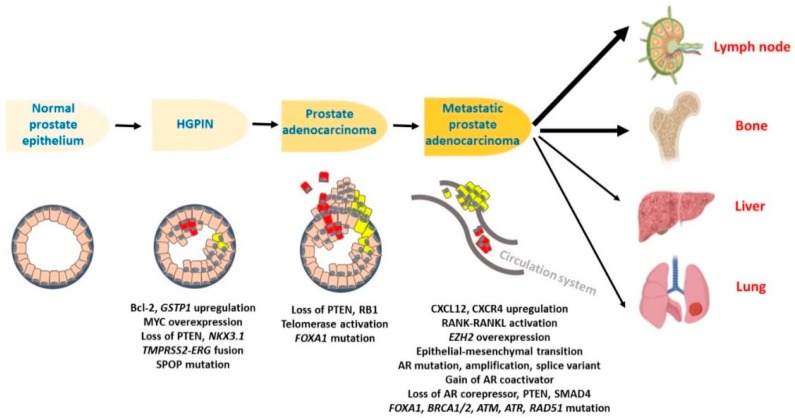
Cellular progression of prostate cancer. Prostate cancer originates from the luminal or basal cells of the normal prostate epithelium. At first, premalignant lesions, referred as prostatic intraepithelial neoplasia (PIN) develop. Only the high-grade PIN (HGPIN) transform into malignant invasive prostate adenocarcinoma and eventually progress to become a metastatic disease spreading to the lymph nodes, bone, liver, and lung via the circulation system. Different molecular alterations have been reported in different stages of prostate cancer progression. In HGPIN, an overexpression of *BCL2*, *GSTP1*, *MYC* and a loss of *PTEN*, *NKX3.1, TMPRSS2-ERG* fusion and *SPOP* mutation are reported. In early stage prostate carcinoma, a loss of tumor suppressor genes such as *PTEN* and *RB1* and overexpression of certain oncogenes with frequent mutations such as *FOXA1* have been reported. During progression to metastatic stage, multiple molecular alterations such as overexpression and/or mutations in *AR*, *ATM*, *ATR*, *RAD51,* and *CXCR4,* and loss of various tumor suppressors such as *SMAD4* has been reported.

Malignant progression of prostate tumors is a multistep process (Figure 1). During early stages, premalignant lesions develop that are referred to as prostatic intraepithelial neoplasia (PIN) [18]. Subsequently, localized invasive lesions emerge, which finally progress to advanced metastatic prostate adenocarcinomas [9]. PIN are classified into low- and high-grade lesions of which only the high-grade PIN (HGPIN), also referred as in situ carcinoma, is largely recognized as a precursor to invasive prostate cancer. The PIN is characterized with the presence of luminal epithelial cells having prominent and enlarged nucleoli and HGPIN share many genetic and molecular similarities with cancer cells. Multiple proliferative abnormalities are also seen quite often in HGPIN as compared to the normal and hyperplastic epithelium. While in the benign epithelium, proliferation occurs in the basal cell compartment, it predominantly occurs on the luminal side of the ducts and acini in HGPIN [19,20]. Overexpression of several oncogenic proteins such as anti-apoptotic BCL2 and the π-class glutathione S-transferase (GSTP1) is reported in the premalignant lesions [21]. Elevated BCL2 expression is suggested to be responsible for the protection of cells from apoptosis during the normal to malignant transformation. GSTP1 is detected in more than 70% of high grade PINs, while its expression is lost in more than 90% of PCa patients possibly due to promoter hypermethylation [21]. Moreover, higher expression of proliferation marker, Ki-67, and decreased expression of a cyclin-dependent kinase inhibitor, p27 (Kip1), is reported in high-grade PIN [21]. Loss of expression of tumor suppressor genes, such as PTEN and NKX3.1, is also reported in the high grade PIN [22]. TMPRSS2-ERG fusion gene is also detected in high-grade PIN lesions, and this molecular rearrangement is suggested to be an early event in the development of prostate cancer [23]. In a separate study, ERG overexpression in adult murine PCa cells is shown to lead to the development of epithelial hyperplasia and focal PIN lesions [24]. Downregulation of miR-34 family, miR-23b, and miR-205 has also been suggested to be important events for the development of premalignant lesions [25]. During the progression from high grade PIN to carcinoma, activation of telomerase, PTEN deletion and loss of RB1 has been suggested to be the critical [22]. Loss of the miR-15A-miR-16-1 cluster on chromosome 13, AR mutation and amplification, overexpression of multiple oncogenes such as CXCR4, EZH2, cMYB, activation of several oncogenic pathways and various mutations including FOXA1, BRACA1/2, and ATM are also reported to correlate with the PCa progression from early stage to metastatic disease.

## 3. Androgen Receptor: Structure and Mechanisms of Action

**Figure 2:**
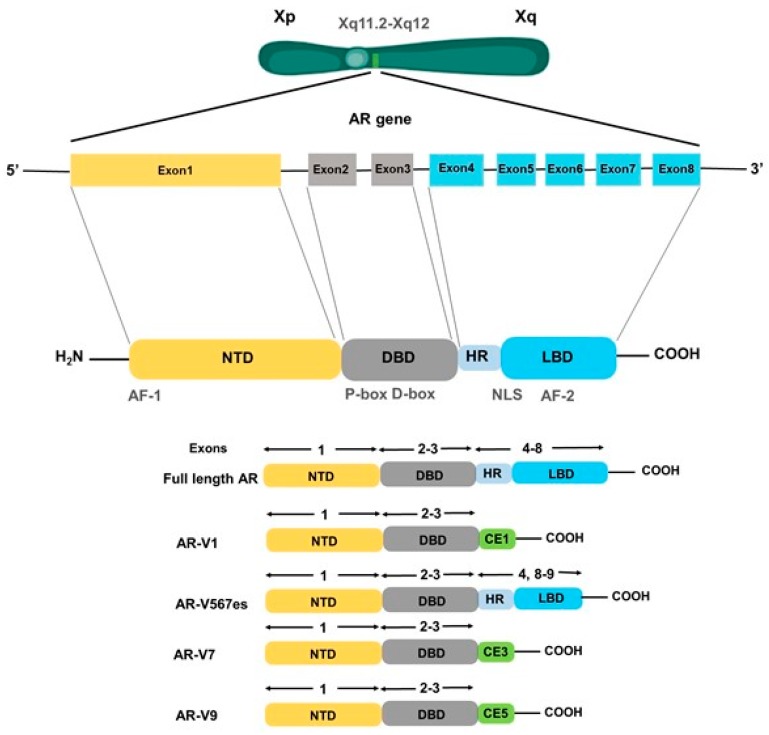
Genomic organization of the *AR* gene and frequently reported androgen receptor (AR) splice variants. The gene encoding for androgen receptor is localized on the long arm of chromosome X in the region Xq11.2-q12. It is comprised of 8 exons that code for the mature androgen receptor protein having three functional domains: N-terminal binding domain (NTD), DNA binding domain (DBD), and a carboxy-terminal ligand binding domain (LBD) that is separated from DBD by a short hinge region (HR). Exon1 encodes for the NTD, exon 2–3 encode for the DBD, exon 4–8 encode for the hinge region and LBD. Several AR splice variants have been reported that show truncation at DBD or LBD. AR-V1 is truncated at the end of exon 3 and contains 19 amino acids from cryptic exon 1 (CE1). AR-V567 is created by skipping of exons 5–7 in the AR mRNA, while AR-V7 has splicing of cryptic exon 3 (CE3) after exons 1–3, and AR-V9 is generated by splicing of cryptic exon 5 (CE5) after exon 3. AR-V1, AR-V7, and AR-V9 all lack LBD.

**Figure 3:**
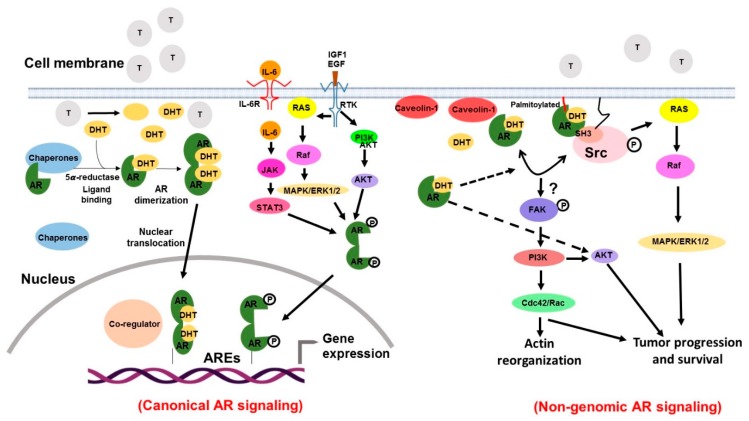
The genomic and non-genomic androgen receptor signaling. Androgen receptor (AR) in an inactivated state remains sequestered in the cytoplasm by chaperone proteins. Binding of androgens to the AR results in the dissociation of chaperon complex that causes a conformational change in the AR followed by its dimerization. AR dimer translocates into the nucleus and binds to target gene promoters/enhancers facilitated through its interactions with co-regulators. It then recruits RNA polymerase-II to initiate gene transcription. AR can also impact cell signaling without directly binding to the gene promoters. In the absence of androgens, various growth factors and cytokines may also activate the AR by regulating multiple signaling pathways. In non-genomic AR signaling, membrane-anchored (through palmitoylation) or membrane-recruited (through Caveolin 1) AR interacts with the SH3 domain of Src leading to its unfolding and activation of the kinase domain. The activated Src then induces Ras-mediated MAPK/ERK signaling. Membrane-anchored AR is also shown to trigger cytoskeletal reorganizations via FAK/PI3K/Cdc24/Rac1 induced signaling.

Androgen receptor (AR) is a nuclear transcription factor and a steroid hormone receptor. The gene encoding for the AR is located on the chromosome Xq11.2-q12, consists of 8 exons [26] and spans 186,587 kb (Figure 2). The structure of AR bears high similarities with other steroid receptors such as progesterone receptor (PR), estrogen receptor (ER), glucocorticoid receptor (GR), and thyroid hormone receptor (TR) [27,28]. It has four domains: a moderately conserved ligand-binding domain (LBD), a highly conserved DNA-binding domain (DBD), a poorly conserved N-terminal domain (NTD), and the hinge region separating the LBD from the DBD. The NTD accounts for more than half of the size of the AR, and is encoded by exon 1. Within the NTD, an Activating factor 1(AF1) region is present, which is considered its primary effector region. The AF1 consists of 2 transcription activation units i.e., Tau-1 (aa 100–370) and Tau-5 (aa 360–485) and both of which are indispensable for the complete transcriptional activity of the AR. Tau-1 contains FQNLF motif (aa 23–27) and Tau-5 contains the WHTLF motif (aa 433–437), which are critical in mediating the ligand-dependent, inter-domain interaction between the NTD and the LBD. NTD-LBD interaction is important for the stability of the AR dimer complex [29]. The NTD also contains a poly-glutamine sequence encoded by a CAG triplet repeat sequence, which begins at codon 58, and CAG repeat length has often been inversely associated with the risk of getting prostate cancer [30]. DNA-binding domain (DBD) is encoded by exons 2 and 3, and comprised of two zinc finger motifs. Exon 2 encodes for the α-helix N-terminal zinc finger, which interacts with the nucleotides located in the hormone response element in the DNA major groove. Exon 3 encodes the second zinc finger containing a conserved D-box motif (ASRND). The selectivity to specific DNA response element is known to be achieved by AR dimerization through the Distal box region that allows the AR to bind to direct repeat half-sites in its promoter. The nuclear localization signal present at the junction between the DBD and the hinge region is important for the nuclear import of the AR [30,31,32]. Ligand binding domain (LBD) is encoded by exons 4–8 and required for the binding of AR to its ligands, testosterone and dihydrotestosterone (DHT). Majority of the AR point mutations in prostate cancer have been identified in the LBD suggesting their importance in pathobiology associated with aberrant AR signaling. Hinge region is a short amino acids sequence that possesses a bipartite ligand-dependent nuclear localization signal (NLS) and helps in the nuclear transport of the AR [33]. Furthermore, several AR splice variants (AR-V1, -V7, and -V9) lacking LBD are also commonly reported (Figure 2). Another splice variant, AR-V567es is also reported in prostate tumor [34,35,36,37]. Studies have documented that even though some AR variants lack LBD, they retain their ability to bind to DNA and promote constitutive gene transcription in the absence of androgens [38,39]. As per the canonical model of AR action, inactive AR resides in the cytoplasm sequestered by multiple chaperones and co-chaperones (such as heat shock proteins, FKBP51, FKBP52, and Cyp40), and cytoskeletal proteins [40]. Ligand binding to the LBD of AR triggers dissociation of chaperone proteins inducing conformational changes that enable AR dimerization and interactions with a cytoskeletal protein, Filamin A to facilitate its nuclear translocation. Nuclear AR binds to the androgen response elements (AREs) in the promoter and enhancer regions of the downstream target genes and forms transcriptional coactivator complexes that remodel the chromatin structure to access the target initiation site. Then, the complex stabilizes the RNA pol II machinery for repeated rounds of transcription. In parallel, ligand-independent activation of AR signaling via growth factors- and cytokines-mediated mechanisms has also been reported. For example, IL-6 binding to the IL-6 receptor causes MAPK- and STAT3- mediated transcriptional activation of AR [41]. Similarly, EGF in the absence of androgen is also shown to transcriptionally activate AR via RAS/RAF/MAPK and PI3K/Akt pathways [42,43,44]. Besides this genomic mode of action of AR, many of the cellular responses of androgens do not require AR-mediated changes in gene expression. It is established that ligand-bound AR can associate with other molecular effectors in the cytoplasm and inner part of the plasma membrane as well to activate cell signaling cascades [45,46]. AR-NTD is shown to interact with the SH3 domain of Src and this interaction results in unfolding of Src and subsequent autophosphorylation through the activation of kinase domain. The activation of Src also results in the elicitation of Ras-mediated MAPK/ERK signaling [47]. Some studies have shown that AR may be localized to the cell membrane via palmitoylation or other membrane proteins such as Caveolin [48,49]. The membrane-bound AR (mAR) can trigger cytoskeletal reorganizations via FAK/Cdc24/Rac1/PI3K and modulate the adhesive and migratory activity of prostate tumor cells [50,51] (Figure 3).

## 4. Evidence Implicating Androgen Receptor Signaling in Prostate Cancer Pathogenesis

It is well established that AR signaling plays a central role in the initiation and progression of prostate cancer. Indeed, the disease progression is closely associated with a relative increase in serum prostate specific antigen (PSA), which is encoded by an androgen-regulated gene, KLK3. Besides, targeting of androgen signaling, although not effective in very long term, provides impressive therapeutic benefit. Even in the resistant prostate tumors, AR signaling is considered to play an active role as evidenced by PSA relapse and other clinical data. Below we describe some common AR aberrations implicating this signaling node in prostate cancer pathogenesis.

### 4.1. AR Mutations

The point mutation of AR in the ligand-binding domain is reported in prostate cancer and more frequently in patients with castration-resistant disease. This mutation leads to the loss of AR specificity to natural agonists. Mutations at codon 701 (L701H) in exon D and at codon 877 (T877A) in exon H are frequent in metastatic tissues [52] and associated with AR sensitivity to the glucocorticoids [53]. T877A is the most frequent mutation and is shown to cause AR activation by estrogens, progestins, and even antiandrogens [54,55,56]. Another point mutation, T878A is associated with resistance to the second-generation AR biosynthesis inhibitor, abiraterone [57], whereas point mutation at 876 (F876L) confers resistance to enzalutamide [57]. Some other LBD missense mutations have been identified such as L702H, W742C, and H875Y and their role in the development of CRPC has been suggested [58,59].

### 4.2. AR Amplification/Overexpression and Alternative Splicing

Overexpression of AR is one of the most common alterations (30–50% patients) reported in castration-resistant prostate cancer (CRPC) [60]. Increased expression of AR hypersensitizes the tumor cells to very low levels of androgens and mediates the agonist to antagonist switching [61] causing resistance to AR targeting agents such as bicalutamide [62]. Moreover, AR amplification has also been detected in circulating tumor cells and cell-free circulating tumor DNA from patients with CRPC [57,60]. Splice variants of AR, such as AR-V1, AR-V567es, AR-V9, and AR-V7 are reported to be overexpressed in CRPC. Among these variants, AR-V7 has been extensively studied and endogenously detected at the protein level [63]. In preclinical studies, Niclosamide, an FDA-approved anti-helminthic drug, has been shown to inhibit protein levels of AR-V7 in CRPC cell lines inducing cell death [64]. Clinical trials assessing the efficacy of cabazitaxel in patients with AR-V7-positive cases have shown promising results [65]. These variants can act independent of ligand-binding and hence confer resistance to ADT, enzalutamide, and abiraterone [36,66,67,68,69].

### 4.3. Altered Expression of AR Co-Regulators

AR co-regulators interact with AR to modulate gene expression. So far, more than 170 co-regulators have been identified that are associated with CRPC [70]. The steroid receptor coactivator (SRC), which is a member of the p160 coactivator family for nuclear hormone receptor, interacts with AR. SRC1 enhances the AR activity under low androgene levels and even enhances the activity of the T877A mutant in CRPC cells [71]. SRC-2 gene is amplified in both primary and therapy-resistant prostate cancer [72]. This amplification results in PI3K activation, lipogenesis and drives metabolic process to promote prostate cancer metastasis [73]. SRC-3 gene expression is also increased in CRPC and aids in tumor cell proliferation and survival [74]. SRC-3 mediates the activation of AKT/mTOR signaling in a steroid-independent manner. Furthermore, AKT/mTOR signaling is demonstrated to be critical for the SRC-3 overexpression-induced PCa cell growth [75]. AR-associated protein 70 (ARA70) is also a coregulator of AR that interacts with the amino- and carboxy-terminal regions of AR and promotes its transcriptional activity [76,77,78].

### 4.4. Androgen Biosynthesis

An important mechanism implicating abnormal AR signaling is intra-tumoral synthesis of androgens, which is mostly reported in CRPC [79,80]. CYP11A1 and CYP17A1, which are the members of cytochrome P450 enzyme family, are overexpressed in CRPC and involved in the synthesis of weak androgens, dehydroepiandrosterone (DHEA) and androstenodione. These weak androgens can be converted into testosterone and DHT through the actions of 3βhydroxysteroid dehydrogenase (HSD3β1, HSD3β2), type 5 17β-HSD (AKR1C3) and 5α-reductase type 2 that are expressed by the prostate tumor cells [81]. In some studies, de novo synthesis of androgens from cholesterol has also been reported in metastatic CRPC [82,83].

## 5. Established and Evolving Therapies Targeting Androgen Receptor Signaling in Prostate Cancer

Since AR signaling is a major driver of prostate cancer growth, its targeting has been exploited for therapeutic benefit since past several decades. In most cases, direct targeting of either the ligand or the receptor has been exploited. In some cases; however, alternative approaches have been evaluated to indirectly suppress AR signaling via targeting of its co-factors. In sections below, we discuss various strategies employed to target this important signaling node along with their success and limitations. (see in Table 1, Table 2, Table 3 and Table 4).

### 5.1. Targeting the Ligand

Physiologically, hypothalamus holds a major control on the biosynthesis and secretion of androgens by testes. Luteinizing hormone-releasing hormone (LHRH) is released from the hypothalamus, which stimulates the pituitary gland to release follicle stimulating hormone (FSH) and luteinizing hormone (LH). LH then acts on the receptors present on the Leydig cells in the testes to enzymatically regulate the conversion of cholesterol to two key intermediates, dehydroepiandrosterone and androstenedione. With the action of enzyme 17-beta-hydroxysteroid dehydrogenase, androstenedione is then further processed and converted to testosterone. FSH acts on the sertoli cells present within the testes and induces the expression of LH receptors. Testosterone can further be processed enzymatically to a highly potent form i.e., dihydrotestosterone (DHT). Therefore, targeting of LHRH via agonists or antagonists has been exploited to achieve lowering of serum androgen levels (Table 1).

#### 5.1.1. LHRH Agonists

LHRH agonists (also referred as LHRH analogs) were introduced in the United States in 1984 for hormonal therapy of prostate cancer [129]. LHRH agonists are synthetic peptide similar to LHRH in structure that interacts with LHRH receptor present on the pituitary gland and inhibit LH production. This results in the decreased stimulation of Leydig cells leading to reduced androgens synthesis. LHRH agonists currently used for the ADT include buserelin, leuprolide (Lupron), goserelin (Zoladex), Histrelin, and triptorelin (Trelstar). Lupron causes a prolonged decrease in the release of FSH and LH due to the continuous stimulation of the anterior pituitary leading to the desensitization of the LHRH receptor and therefore, causing the suppression of androgen synthesis [130]. Some of the drawbacks of LHRH agonists are their high costs, impotence, hot flashes and libido loss in treated patients [131]. Moreover, it has also been cautioned that the testosterone surge after initial administration of LHRH agonists can cause tumor flare in majority of the patients with advanced disease [132,133]. To minimize these harmful side effects, various approaches have been tested that include combined treatment with other androgen signaling targeting agents such as leuprolide and bicalutamide [134].

#### 5.1.2. LHRH Antagonists

LHRH antagonists control testosterone levels better and faster than agonists [135]. They directly inhibit the LHRH-receptor interaction resulting in the immediate stopping of the LH and quick suppression of testosterone and DHT production [136,137]. Abarelix was the first LHRH antagonist that was tested against advanced PCa. Findings from the Phase II and III clinical trials established that Abarelix does not produce some of the harmful effects of the LHRH agonist therapy such as a surge in serum testosterone [138]. Abarelix produced rapid medical castration by depleting FSH and LH levels more efficiently and quickly as compared to the LHRH agonists [139,140]. Moreover, it had the comparable safety profile to that reported for LHRH agonist monotherapy in Phase III clinical studies [140]. In some patients; however, Abarelix was found to cause the release of histamine that led to systemic allergic reactions. Cetrorelix and ganirelix, two other LHRH antagonists, have also been tested for their efficacy and reported to achieve testosterone suppression in prostate cancer patients in clinics [141]. In 2008, a new generation LHRH antagonist, Degarelix, was developed and got FDA-approval for advanced PCa [142]. Degarelix is more potent and long-acting LHRH antagonist that effectively suppress testosterone levels in patients with PCa as reported in a phase II clinical trial [136]. Moreover, it did not cause any events of testosterone surge or immediate or late onset systemic allergic reactions [143]. In a study comparing the efficacy of Degarelix alone or in a combination therapy with LHRH agonist, Goserelin, and bicalutamide, it was reported that the former produced greater urinary tract symptom relief and improved life in PCa patients [144].

#### 5.1.3. Androgen Synthesis Inhibitor

After it was established that intra-tumoral androgen synthesis and adrenal androgens can be a cause of failure of castration therapy [145,146] efforts began to develop novel strategies for targeting of the involved biosynthetic enzymes. Ketokonazole, is a synthetic antifungal drug, which is soldoff-label and also being used as second-line therapy against mCRPC treatment since 1984 [147]. This drug inhibits the enzyme, CYP171A, and blocks the gonadal and adrenal steroidogenesis as a non-specific cytochrome P450 (CYP450) inhibitor [148,149]. Ketokonazole treatment showed the decrease of PSA level in patients [150,151] especially in combination with hydrocortisone [152] and docetaxel [153]. However, no studies have shown the improvement in the overall survival of patients, whereas the adverse effects such as gastrointestinal intolerance, followed by fatigue, liver function abnormalities, and skin changes have been reported [154]. Abiraterone acetate is the first drug approved by the FDA that reduces both adrenal and intra-tumoral androgen synthesis by inhibiting cytochrome p450 (CYP) 17′s 17α-hydroxylase and 17,20-lyase activities. In a phase I clinical trial, CYP17 blockade by abiraterone acetate was shown to suppress serum testosterone, downstream androgenic steroids, and estradiol in all the enrolled patients. Moreover, it exhibited significant antitumor activity in CRPC patients [155]. Later, it was discovered that Abiraterone led to the elevation of the adrenocorticotropic hormone and therefore, to suppress this adverse effect, low-dose co-administration of prednisone was recommended [156]. In a recent study, potent clinical activity of Abiraterone acetate has been suggested, in part, to its active metabolite Δ (4)-abiraterone (D4A). D4A inhibits several enzymes of steroid biosynthetic pathway including CYP17A1, 3βHSD, and SRD5A, which are required for DHT synthesis. Moreover, it is shown to also cause efficient antagonization of the AR at a comparable level to that reported for the potent antagonist, enzalutamide, in a preclinical mouse model of PCa because its steroid A and B rings are identical to testosterone [157,158].

In recent years, other drugs targeting androgen biosynthesis have also been developed. Orteronel (TAK-700), a new drug, is shown to be five times more selective than abiraterone in inhibiting the 17,20-lyase activity of monkey and human CYP17A1 and it drastically reduced serum androgen levels in monkeys [98]. A phase II study evaluated its response in CRPC patients and reported lower levels of circulating androgens and tumor cells in treated patients [159]. The treatment with Orteronel prolonged the progression-free survival from 8.7 to 13.8 months; however, no effect on the overall survival was reported [160]. Another small-molecule CYP17A1 inhibitor, VT-464, also selectively inhibits the 17,20-lyase activity in the abiraterone- and enzalutamide-resistant PCa cell line model [161]. Finasteride, is a inhibitor of 5α-reductase enzyme that blocks the conversion of testosterone to DHT and thus decreases the DHT synthesis in men [99].

### 5.2. Targeting the Receptor

Having observed bypass mechanisms and some adverse effects of ligand-targeted therapy, efforts were initiated to develop novel therapeutic strategies that target the AR directly. Most of the approaches that have been evaluated involve the use of steroidal or non-steroidal anti-androgens. These compounds inhibit the binding of the ligand to the AR and have met with mixed success and failure (Table 2). 

#### 5.2.1. Steroidal Anti-Androgens

The development of steroidal antiandrogens began in 1962. Among the important steroidal antiandrogens are progestogens, cyproterone acetate (CPA), megestrol acetate (MA), dienogest, and chlormadinone acetate (CA). Of these, CPA was the most commonly used drug in the clinics for the treatment of PCa. CPA is derived from hydroxyprogesterone, which has greater binding affinity for the AR as compared to the first generation non-steroidal antiandrogens that are discussed later [162,163]. CPA not only functions as an antiandrogen, but also as anti-gonadotropin as well since it competes with gonadotropin for the binding to its receptor (GnR) [164]. The use of CPA in clinics has; however, been discontinued after it was shown to have poor efficacy and provided no survival benefit in later clinical trial studies either as a montherapy or when combined with surgical castration [165,166]. In addition, steroidal antiandrogens were also associated with adverse events such as hepatotoxicity and cardiovascular side effects although in some cases, a combined treatment of steroidal antiandrogens with LHRH treatment reduced gynecomastia and hot flashes [167]. 

#### 5.2.2. Non-Steroidal Anti-Androgens

Non-steroidal agents have narrower target range and thus exhibit fewer side effects compared to the steroidal anti-androgens. Bicalutamide, Flutamide and Nilutamide are the first-generation nonsteroidal anti-androgens. Bicalutamide binds to the allosteric site on the AR inducing a conformational change in the co-activator binding site and thus interferes with its transcriptional activity. A reduction in AR expression is also observed following bicalutamide treatment, but it is transient. It is also shown that bicalutamide, instead of working as antagonist, produces agonistic action as well in some CRPC [168]. Flutamide interferes AR signaling by blocking the ligand binding to the AR. In clinical studies, flutamide as monotherapy showed potency in patients that had not undergone any prior treatment. Flutamide has also been used in combination with LHRH agonist as initial therapy for metastatic PCa [169]. Nilutamide functions via competing with testosterone and DHT for binding to the AR. Nilutamide is recommended for use in stage D2 metastatic PCa along with castration. In post-castration progressive PCa, Nilutamide is shown to delay PSA progression [170]. Enzalutamide and Apalutamide are the second-generation non-steroidal antiandrogens. Enzalutamide has high affinity for the LBD of AR and blocks its nuclear translocation. It is approved for the treatment of patients with metastatic CRPC in both pre- and post-chemotherapy settings. In a phase III, double-blinded, clinical study, enzalutamide prolonged the survival of patients with mCRPC after docetaxel treatment. Some adverse effects have also been reported for Enzalutamide including fatigue, diarrhea, hot flashes, and seizures as it crosses the blood–brain barrier and acts on γ-aminobutyric acid receptors [171]. Apalutamide also works through a similar mode of action as enzalutamide, but has a higher therapeutic index due to greater AR affinity [172,173]. Both apalutamide and Enzalutamide are FDA approved for mCRPC. Apalutamide was initially approved for non-metastatic CRPC (PSA progression) in 2008, but in 2019 also received the approval for metastatic disease as well. AZD3514 is another second generation oral selective androgen receptor inhibitor that acts slightly differently as it not only inhibits the nuclear translocation of AR but also decreases the rate of AR synthesis [109].

AZD3514 demonstrated anti-tumor activity in HID28 mouse model of CRPC [109]. Moreover, it has shown good potency in clinical studies as well, but only moderate anti-tumor activity was reported in patients with metastatic CRPC [174]. ODM-201, currently marketed as Darolutamide recently received FDA approval for the treatment of non-metastatic CRPC. Some consider it as a third-generation anti-androgen, which is not affected by the F876L AR mutation that is critical for the resistance to enzalutamide and apalutamide [110]. In the phase I/II clinical trial, Darolutamide has demonstrated promising antitumor activity in patients with metastatic CRPC [175].

### 5.3. Targeting the AR Interaction with Co-Regulators

AR signaling can also be regulated indirectly via the targeting of co-receptors and other chaperon proteins (Table 3). Steroid receptor coactivators (SRC) is a large class of proteins that normally interact with steroid receptors in a ligand-dependent manner and enhance their transactivation [176,177]. Pharmacological inhibition of SRC using MCB-613, a small-molecule inhibitor, is shown to selectively kill PCa cells and inhibit tumor growth in vivo [111]. Unfortunately, advanced stage PCas often exhibit the presence of AR splice variants, which support resistance to castration therapies as they lack the ligand-binding domain [63,178]. The most frequent variant is AR-V7, which is resistant to abiraterone and enzalutamide [66]. Interaction of an AR coactivator Vav3 with AR-V7 is suggested to be a mechanism for CRPC where it promotes the ligand-independent transcriptional activity of AR-V7 [179,180]. Accordingly, disruption of this interaction is shown to suppress the proliferation and aggressive phenotype of CRPC cells [181]. Another AR variant coactivator is DBC1 protein, which directly interacts with AR-V7, and its inhibition results in AR-V7 degradation and reduced tumor growth [182]. Studies have also shown that drugs such as galeterone that target AR stability or the N-terminal domain may be useful against these splice variants as well. EPI-001 is one such pharmacologically active compound that disrupts transactivation and cofactor recruitment at the N-terminal domain and thus inhibits the activity of both wild-type and splice variant forms of AR [183]. A more potent derivative of EPI-001, EPI-506, has been developed, which is referred as the first-generation androgen receptor N-terminal domain inhibitor. EPI-506 produced promising outcome in PCa patients that expressed AR splice variants. AR activation function-1 (AF-1) region, which is present in N-terminal domain of both full-length and variant AR, can also be targeted by another drug, EPI-002, and its potency in treating CRPC has been suggested in preclinical studies [184]. 

Heat shock proteins (HSPs) also play a significant role in AR signaling axis [185]. HSP 90 is shown to stabilize the AR in the cytoplasm in a multi-chaperone protein complex. Upon ligand binding, it dissociates from the AR facilitating AR nuclear translocation [186,187]. Several inhibitors have been developed that target either HSPs or their co-chaperones to modulate AR transcriptional activity [188]. Hsp90 inhibitor, 17-allylamino-17-demethoxygeldanamycin (17-AAG), has been reported to prevent the ligand-independent nuclear translocation of AR in CRPC cells [189]. In addition, 17-AAG delayed the castration resistance progression of LuCaP35 xenograft tumors and prolonged the survival of tumor-bearing mice [116]. However, it failed to show the desired response in a phase II clinical trial in patients with hormone-refractory metastatic PCa. Due to severe toxicity and insufficient PSA response, the trial was terminated at the end of first stage [190]. Methoxychalcones that stabilize the Hsp90-AR-complex have also shown some promise in laboratory studies, but require further validation [117]. Onalespib, a second generation HSP90 inhibitor, downregulates AR-V7 mRNA levels without affecting total AR transcript levels in PCa [118]. However, it showed only marginal efficacy in clinical studies in combination with Abiraterone Acetate (AA) and prednisone/prednisolone (P) in PCa patients that progressed after AA/P treatment [191].

### 5.4. Targeting by AR Signaling by the Natural Agents

Bioactive natural products derived from fruits, vegetables, herbs, marine sponges, and edible plants are considered potent chemo-preventive and/or -therapeutic agents and can be used either alone or as adjuvants. Several natural agents have shown good efficacy against PCa in laboratory and preclinical studies and many of them target AR signaling (Table 4). Several studies have shown that EGCG, a green tea component, inhibits the enzyme 5-α-reductase that transforms testosterone into 5-α-dihydrotestosterone and thus regulates androgen activity [192,193]. It is also shown that EGCG inhibits the expression and nuclear translocation of AR in PCa cells by physically interacting with its ligand-binding domain [194]. Fujita et al. demonstrated that the extracts from an edible mushroom, Ganoderma lucidum, could potently inhibit 5α-reductase activity and reduced androgen synthesis [195]. Glycyrrhetinic acid (GA), a bioactive metabolite present in licorice, also targets two key enzymes involved in androgen synthesis, 17,20-lyase required for the conversion of 17-hydroxyprogesterone to androstenedione, and 17β-hydroxysteroid dehydrogenase required for the conversion of androstenedione to testosterone [196]. Curcumin is an isoflavone isolated from the roots of the plant Curcumin longa and has been known for its anti-inflammatory and anticancer activities since ancient times. Ohtsu and colleagues developed curcumin analogs and demonstrated their superior anti-androgenic activities as compared to the anti-androgen (hydroxyflutamide) used in clinics [197]. Studies have also shown the potent activities of curcumin analogs in the inhibition of testosterone/ DHT-induced AR activity and growth suppression of CWR-22Rv1 and LNCaP cells [198,199]. Natural compounds targeting the amino-terminus of the AR have also been identified. Marine sponge-derived small molecule, EPI-001, inhibits both the androgen-dependent and androgen-independent activation of AR via its direct binding to the AF1 region of the NTD in PCa cells carrying full length or the truncated form of the AR lacking the LBD [114,183,200]. Moreover, EPI-001 halted the androgen-induced proliferation of CRPC in xenografts mouse model [114]. Meimetis et al. 2012 demonstrated that Niphatenones, glycerol ethers from the sponge Niphates digitalis, interfered with the AR transcriptional activity in LNCaP cells via covalent binding to the AF1 region of the NTD [123]. Similarly, sintokamides A to E derived from the marine sponge Dysidea sp. suppressed the AR activity in LNCaP cells via inhibiting N-terminus transactivation of the AR [201]. Selenium, predominantly found in fish, meat, eggs, and grains, is reported to regulate AR as well as other genes involved in androgen regulation [202]. Christensen and coworkers reported that selenium inhibited NF-κB and regulated the expression of multiple genes including AR in PCa cells [203]. Quercetin, a flavanol pigment present in apples and onions, is reported to act as Hsp70 inhibitor and its anti-tumor potency is demonstrated in AR-V7 expressing PCa cells [204]. Berberine, a phytochemical isolated from Berberis plant is shown to inhibit the interaction of Hsp90 with AR leading to the proteasomal degradation of AR [205]. Hydroxytyrosol (HT), predominantly found in olives, have been shown to possess anti-cancer activity in PCa by inhibiting oncogenic signaling pathways and inhibiting the expression of AR in laboratory studies [128].

## 6. Conclusions and Future Perspective

Therapeutic landscape of PCa has improved significantly particularly over the last decade or so. This has resulted in longer life for patients benefitting from the widespread use of new generation drugs and combination therapies. We have gathered new molecular insights from failed therapies and laboratory studies that have helped us made newer advances. Significant efforts are now being focused on directly targeting the AR especially after the reports suggesting a critical role of constitutively active AR variants in castration resistance that allows persistent disease progression. There is also an increasing realization that therapy-resistance is often multifactorial and PCa is a highly heterogeneous disease. A number of studies have suggested the involvement of multiple oncogenic signaling pathways other than AR that can either compensate for the loss of AR signaling or contribute to its aberrant activation, especially in advanced stages. Of note, overexpression of HER2/neu and IGF-1 is shown to activate AR signaling [206]. Similarly, MAP Kinase pathway contributes to the increase in PSA under androgen-independent conditions [207]. A crosstalk between IL-6 and AR signaling has also been reported [41,208]. Findings from our lab and elsewhere have also implicated downregulation of PP2A signaling in castration-resistance of PCa [209,210,211]. We also demonstrated a role of MYB, an oncogenic transcription factor, in androgen deprivation–resistant growth of PCa cells [212]. In some reports, a role of estrogen and glucocorticoid signaling in castration resistance is also reported [213,214,215,216]. We anticipate that novel therapies based on these resistance mechanisms will also be developed not in very distant future. We also expect that upcoming trials will test and optimize the sequencing and/or dosing of combination regimens utilizing currently available androgen signaling targeted drugs. Altogether, available and emerging molecular and mechanistic information, and ongoing clinical trials give us hope that new treatment options will continue to become available for oncologists to better manage this dreaded malignancy.

## Figures and Tables

**Table 1:** List of compounds targeting the androgen synthesis directly or indirectly and their mechanisms of action (* compounds that are/were in clinical practice, ** compounds that show promise in preclinical studies, but have not yet been evaluated in clinical trials or have yet brought to clinical practice).

LHRH Agonist		
Compound	Mechanism of Action	Ref
Buserelin *	Binds to and desensitizes LHRH receptor, and thus, reduces the level of testosterone. Not available in US, but marketed elsewhere in the world	[84,85]
Leuprolide *	Binds to LHRH receptor and acts as an inhibitor of gonadotropin secretion. The prolonged exposure of the agonist decreases the secretion of LH, FSH, and testosterone.	[86]
Goserelin *	Binds to and activates the LHRH receptor to inhibit the release of pituitary gonadotropin and thus reduces the testosterone level.	[87,88]
Histrelin *	Binds to LHRH receptor and acts as an inhibitor of gonadotropin secretion. The continuous administration of this agonist reduces the levels of LH, FSH and testosterone. This is marketed as Vantas.	[89,90]
Triptorelin *	Binds to LHRH receptor and whose prolonged exposure is shown to decrease the secretion of LH, FSH and testosterone.	[91,92]
**LHRH Antagonist**		
Abarelix *	Binds to LHRH receptor and acts as a potent inhibitor without initial testosterone surge. Currently available in Germany, but was withdrawn in the US in 2005.	[93]
Cetrorelix **	Binds to the LHRH receptor and inhibits the secretion of LH and FSH. Not yet approved for prostate cancer, but indicated for the inhibition of premature LH surges in women undergoing controlled ovarian stimulation.	[94]
Ganirelix **	Binds to and prevents the LHRH receptor from LHRH binding (with no initial testosterone surge) and thus reducing the release of gonadotropin and testosterone. Not yet approved for prostate cancer, but indicated for the inhibition of premature LH surges in women undergoing controlled ovarian stimulation	[95]
Degarelix *	Binds to and prevents the LHRH receptor from LHRH binding (with no initial testosterone surge) thus reducing the release of LH, FSH, and testosterone.	[96]
**Androgen Synthesis Inhibitor**		
Abiraterone *	Covalently binds to and selectively inhibits the androgen biosynthesis enzyme, CYP17A, in an irreversible manner and hence, reduces the level of testosterone and other androgens.	[97]
**Orteronel** (TAK-700) *	Inhibits the androgen biosynthesis enzyme, CYP17A, and thus reduces the level of testosterone.	[98]
Finasteride *	Inhibits the synthesis of DHT from testosterone.	[99]

**Table 2:** List of compounds targeting androgen receptor, and their mechanisms of action (* compounds that are/were in clinical practice, ** compounds that show promise in preclinical studies, but have not yet been evaluated in clinical trials or have yet brought to clinical practice).

Anti-Androgen: Steroidal		
Compound	Mechanism of Action	Ref
Cyproterone acetate *	Not only functions as an anti-androgen but also possesses potent anti-gonadotropic activity that results in rapid suppression of serum testosterone. The use in clinics has been discontinued	[100]
Megestrol acetate *	Exerts its effects through various mechanisms. Primarily, it acts as an anti-androgen, but can also inhibit 5-alpha reductase and LH release.	[101]
Dienogest **	Binds to and blocks the binding of androgens to the androgen receptor	[102]
**Galeterone** (TOK-001) *	It possesses a dual mechanism of action, acting as both as an anti-androgen and as a CYP17A1 inhibitor suppressing the biosynthesis of androgen	[103]
Chlormadinone acetate *	Acts as a partial antagonist of AR. Also reduces the activity of 5α-reductase and thus inhibiting androgen production and signaling Not approved in US.	[104]
**Anti-Androgen: Non-Steroidal**		
**First Generation Inhibitor**		
Bicalutamide *	Binds to the allosteric site on the AR, induces a conformational change in the co-activator binding site and thus interferes with its transcriptional activity	[105]
Flutamide *	Competitively binds to AR and inhibits the binding of androgen to AR.	[106]
Nilutamide *	Competitively binds to AR and inhibits the binding of androgen to AR.	[107]
**Second Generation Inhibitor**		
Enzalutamide (MDV 3100) *	Selectively binds to AR with high affinity and blocks the nuclear translocation of AR, inhibits the recruitment of coactivator and AR DNA binding.	[108]
Apalutamide (ARN-509) *	Competitively binds to AR with high affinity, reduces the binding of androgen to AR, and inhibits the nuclear translocation of AR.	[109]
AZD3514 **	Binds to the LBD of AR and thus inhibits the ligand-driven nuclear AR translocation.	[110]
Darolutamide (ODM201) *	Binds to AR with high affinity and reduces the binding of androgen to AR leading to inhibition of the nuclear translocation of AR. Some consider it as a “third generation anti-androgen” as its potency is not affected by the *F876L AR* mutation that is considered critical for the resistance to Enzalutamide and Apalutamide.	[111]

**Table 3:** List of compounds that interferes with the interaction of AR with co-regulators/co-factors, and their mechanisms of action (* compounds that are/were in clinical practice, ** compounds that show promise in preclinical studies, but have not yet been evaluated in clinical trials or have yet brought to clinical practice), *** compounds that showed promise in preclinical studies, but failed in clinical trial,

Targeting the Binding of AR and Co-Regulators		
Compound	Mechanism of Action	Ref
MCB-613 **	The inhibitor of NTD that interacts with AF-1 region and inhibits AR activation and AR-mediated signaling pathway.	[112]
EPI-001 ***	Selectively binds AF-1 domain of the androgen receptor and thus represses the transcriptional activity of AR.	[113]
**Ralaniten** (**EPI-506**) **	The derivative of EPI-001, which acts as an inhibitor of NTD that interact with AF-1 region leading to the inhibition of AR activation and AR-mediated signaling pathway.	[114]
EPI-002 **	The stereoisomer of EPI-001, which has the potency to disrupt the NTD of AR and inhibits the transcriptional activity of AR.	[115]
**Targeting AR Co-Factor**(**s**)		
17-AAG ***	Inhibits HSP90 and the ligand-independent nuclear localization of AR	[116]
Methoxychalcones **	Stabilizes AR-Heat shock protein complex and thus prevents AR dimerization.	[117]
Onalespib *	Inhibits HSP90 leading to the degradation of client proteins including AR.	[118]

**Table 4:** List of natural compounds that target AR signaling and their underlying mechanisms of action. All of these compounds have shown promise in preclinical studies, but have not yet been evaluated in clinical trials or have yet brought to clinical practice.

Compound	Mechanism of Action	Ref
**Green tea extract (EGCG)**	The polyphenol in green tea extract inhibits 5α-reductase activity thus impedes the androgen synthesis.	[119]
**Mushroom extract**	The triterpenoid compound in the mushroom extract inhibits 5α-reductase activity and thus impedes the androgen synthesis.	[120]
**Glycyrrhetinic acid**	Targets 17,20-lyase enzyme and thus decreases the testosterone level.	[121]
**Curcumin**	Curcumin analogs function as androgen antagonists, inhibit testosterone-, DHT-induced AR activity.	[122]
**Niphatenones**	The glycerol ethers from the sponge *Niphates digitalis,* which covalently binds to the AF1 region of the NTD.	[123]
**Sintokamides**	The derivative of the marine sponge *Dysidea sp* that binds to N-terminal domain of AR and thus, suppressed the AR activity.	[124]
**Selenium**	Regulates AR gene expression.	[125]
**Quercetin**	The flavonol pigment in onion and apple acts as the HSP70 inhibitor and induces the AR degradation.	[126]
**Berberine**	The plant phytochemical, which acts as the HSP70 inhibitor and thus induces the AR degradation.	[127]
**Hydroxytyrosol**	Inhibits AR expression.	[128]
